# Signalling lymphocyte activation molecule family member 9 is found on select subsets of antigen‐presenting cells and promotes resistance to *Salmonella* infection

**DOI:** 10.1111/imm.13169

**Published:** 2020-01-28

**Authors:** Timothy J. Wilson, Simon Clare, Joseph Mikulin, Christopher M. Johnson, Katherine Harcourt, Paul A. Lyons, Gordon Dougan, Kenneth G. C. Smith

**Affiliations:** ^1^ Department of Microbiology Miami University Oxford OH USA; ^2^ Department of Medicine Cambridge Institute for Medical Research University of Cambridge Cambridge UK; ^3^ Wellcome Trust Sanger Institute Hinxton Cambridge UK; ^4^ MRC Laboratory of Molecular Biology Cambridge UK; ^5^ Cambridge Institute for Therapeutic Immunology and Infectious Disease Department of Medicine University of Cambridge Cambridge UK

**Keywords:** dendritic cells, inflammation, mononuclear phagocytes, *Salmonella*, SLAMF9

## Abstract

Signalling lymphocyte activation molecule family member 9 (SLAMF9) is an orphan receptor of the CD2/SLAM family of leucocyte surface proteins. Examination of SLAMF9 expression and function indicates that SLAMF9 promotes inflammation by specialized subsets of antigen‐presenting cells. Within healthy liver and circulating mouse peripheral blood mononuclear cells, SLAMF9 is expressed on CD11b^+^, Ly6C^−^, CD11c^low^, F4/80^low^, MHC‐II^+^, CX_3_CR1^+^ mononuclear phagocytes as well as plasmacytoid dendritic cells. In addition, SLAMF9 can be found on peritoneal B1 cells and small (F4/80^low^), but not large (F4/80^high^), peritoneal macrophages. Upon systemic challenge with *Salmonella enterica* Typhimurium, *Slamf9^−/−^* mice were impaired in their ability to clear the infection from the liver. In humans, SLAMF9 is up‐regulated upon differentiation of monocytes into macrophages, and lipopolysaccharide stimulation of PMA‐differentiated, SLAMF9 knockdown THP‐1 cells showed an essential role of SLAMF9 in production of granulocyte–macrophage colony‐stimulating factor, tumour necrosis factor‐*α*, and interleukin‐1*β*. Taken together, these data implicate SLAMF9 in the initiation of inflammation and clearance of bacterial infection.

## Graphical abstract

Signalling lymphocyte activation molecule family member 9 (SLAMF9) is a cell surface receptor found on specialized subsets of mononuclear phagocytes. Deficiencies in SLAMF9 expression lead to altered pro‐inflammatory cytokine production in human cells and reduced capacity to clear *Salmonella* infection in mice.

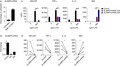

**Abbreviations**
cDC: conventional dendritic cell
ELISA: enzyme‐linked immunosorbent assay
GM‐CSF: granulocyte–macrophage colony‐stimulating factor
ITSM: immunoreceptor tyrosine‐based switch motif
LPS: lipopolysaccharide
M‐CSF: macrophage colony‐stimulating factor
pDC: plasmacytoid dendritic cell
PMA: phorbol‐12‐myristate‐13‐acetate

## Introduction

SLAM family receptors are a family of cell surface proteins with differential expression across all leucocyte subtypes. In humans and mice, they comprise nine members based on protein ectodomain structure and gene phylogeny; SLAM, CD48, Ly9, 2B4, CD84, NTB‐A, CRACC, BLAME and Signalling lymphocyte activation molecule family member 9 (SLAMF9). Of these, six contain one or more cytosolic immunoreceptor tyrosine‐based switch motifs (ITSMs) capable of interacting with the signalling adapter proteins SAP and EAT‐2.^1^, ^2^ Recruitment of these adapters by SLAM family receptors promotes stable intercellular adhesion and cellular effector function,^3^ whereas ITSM signalling in the absence of these adapters can inhibit cellular activation.^4^, ^5^, ^6^, ^7^, ^8^

The best characterized functions of SLAM family receptors are their contributions to intercellular adhesion,^3^, ^9^, ^10^ leucocyte migration^11^ and cellular cytotoxicity.^7^, ^8^, ^12^, ^13^, ^14^, ^15^ In addition to these functions, SLAM family receptors have been directly implicated in pathogen recognition and clearance by macrophages. For example, SLAM contributes to recognition of Gram‐negative bacteria,^16^ and BLAME regulates the production of reactive oxygen species.^17^

The mRNA and amino acid sequences of SLAMF9 were first reported in 2001,^18^, ^19^, ^20^ but the protein has remained minimally characterized since then. SLAMF9 is structurally similar to the other SLAM family receptors. It comprises an N‐terminal immunoglobulin V‐type domain, a C2‐type immunoglobulin superfamily domain, and a transmembrane domain. In contrast to the SLAM family receptors that have long cytoplasmic tails with multiple ITSMs, SLAMF9 has a short, lysine‐ and arginine‐rich cytoplasmic domain. Although the expression of SLAMF9 in mice and humans has remained largely undefined, recent studies have shown expression of SLAMF9 among hepatic macrophages and tumour‐associated macrophages and have implicated SLAMF9 in macrophage responses to lipopolysaccharide (LPS) and the regulation of Toll‐like receptor 4 (TLR4) expression.^21^, ^22^, ^23^ In this study, we use novel monoclonal and polyclonal antibodies to define the expression patterns of SLAMF9 in human peripheral blood mononuclear cells (PBMCs) and mouse circulating and tissue‐resident leucocytes. We show that SLAMF9 differentially regulates pro‐inflammatory cytokine production in response to LPS, and that SLAMF9 is necessary for clearance of *Salmonella* from the liver during systemic infection.

## Materials and methods

#### Generation of SLAMF9‐specific antibodies

Polyclonal rabbit antisera were raised against mouse SLAMF9 C‐terminal peptide RVRKLKRNRIKLRKKGKSG coupled to keyhole‐limpet haemocyanin by Pacific Immunology (Ramona, CA). Peptide‐specific antibodies from serum 9318 were then affinity‐purified by liquid chromatography over peptide‐coupled agarose. Monoclonal anti‐mouse SLAMF9 (M349) was generated by immunizing *Slamf9^−/−^* C57BL/6N mice intraperitoneally twice with soluble mouse SLAMF9‐CD4‐His fusion protein in alum, and once with soluble SLAMF9 in the absence of adjuvant. Three days after the final immunization, splenocytes were fused with SP2/0 myeloma cells and placed under hypoxanthine‐aminopterin‐thymidine selection. Supernatants from 480 of the resulting clones were screened for reactivity by enzyme‐linked immunosorbent assay (ELISA) and by flow cytometry on SLAMF9‐transfected HEK‐293T cells and mouse bone‐marrow‐derived macrophages. Thirty‐two clones were selected for isotyping and further screening. Clone M349 (ms IgG1‐*κ*) was selected for its specificity and reactivity against wild‐type but not *Slamf9^−/−^* bone‐marrow‐derived macrophages by flow cytometry. Monoclonal anti‐human SLAMF9 (FC2; ms IgG1) was generated in a manner similar to M349. Briefly, C57BL/6N mice were immunized twice by intraperitoneal injection with soluble human SLAMF9‐huIgG1‐Fc fusion protein (R&D Systems, Minneapolis, MN) in alum and once in the absence of adjuvant. After fusion, 736 hybridoma clones were screened by ELISA against the antigen as well as human IgG1. Thirteen clones were found to be specifically reactive to the SLAMF9‐Fc fusion protein and not human IgG1. These 13 clones were screened by flow cytometry on human SLAMF9‐transfected HEK‐293T cells. Of these, four (FC2, FC57, FC203 and FC354) were found to recognize human SLAMF9 on the surface of transfected 293T cells, but not untransfected controls. Clones FC2 and FC203 were found to have superior signal‐to‐noise characteristics, and FC2 was expanded for purification and characterization. Direct conjugation of antibodies with biotin or Alexa Fluor 647 was performed using EZ‐Link NHS‐Sulfo‐LC‐Biotin and Alexa Fluor 647 NHS Ester (Thermo Fisher, Waltham, MA) respectively according to the manufacturer's instructions.

#### Generation of *Slamf9^−/−^* mice

*Slamf9^−/−^* mice (C57BL/6N‐A^tm1Brd^ Slamf9^tm1a(EUCOMM)Wtsi^/WtsiOulu) were generated by homologous recombination at the Wellcome Trust Sanger Institute according to previously reported protocols.^24^, ^25^ Knockout‐first (tm1a) alleles were used for most studies reported in this manuscript. Replication of *S*. Typhimurium infection and tissue expression of mouse SLAMF9 were performed using the reporter tagged deletion (tm1b) allelic form. The absence of SLAMF9 protein in both targeted alleles was confirmed by flow cytometry and Western blotting. Routine genotyping of mice was performed using the following primer sets: 5′‐CAGCTTGTGTTTCCACAGCC ‐ forward; 5′‐ATCAAGGATCTGGGAGGGG ‐ wild‐type reverse; 5′‐TCGTGGTATCGTTATGCGCC ‐ cassette reverse. All animal procedures were performed according to UK Home Office regulations and Miami University Institutional Animal Care and Use Committee‐approved protocols.

#### Analysis of SLAMF9 expression by flow cytometry

Human PBMCs were isolated from peripheral blood or leucocyte cones of healthy donors by density gradient centrifugation using Histopaque 1077 (Sigma, St Louis, MO). Ethical approval was obtained from the NRES Committee, East of England‐Cambridge Central. Cells were stained with the following antibodies from Miltenyi Biotech (Bergisch Gladbach, Germany), BD Biosciences (Franklin Lakes, NJ), eBioscience (San Diego, CA), and Sigma: CD14‐FITC (TUK4), CD3 (OKT3), CD16 (B73·1), CD19‐V450 (HIB19), and IgG1 isotype control (MOPC21). Anti‐SLAMF9 (FC2) was generated in our laboratory for this study. Cell viability was determined using the Fixable Blue Dead Cell Stain Kit (Thermo Fisher) or Zombie Aqua Dye (BioLegend, San Diego, CA). For analysis of mouse tissues, peritoneal exudate cells were removed by flushing the peritoneal cavity with phosphate‐buffered saline, 1 mm EDTA, and spleens were removed from mice and mechanically disaggregated. Livers were perfused through the portal vein with Hanks' balanced salt solution (HBSS) containing 0·5 mg/ml collagenase D (Roche, Basel, Switzerland). Liver tissue was then macerated and incubated in HBSS/collagenase for 45 min and passed through a 70‐μm cell strainer. Hepatocytes were removed by two brief centrifugation steps at 50×, followed by density gradient centrifugation using Histopaque 1119 (Sigma). Resulting leucocytes were stained with the following antibodies from BD Biosciences, BioLegend, eBioscience, and this study: CD45.2 (104), CD11c (HL3), CD11b (M1/70), CD8α (53–6·7), CX_3_CR1 (SAO11F11), Ly6C (AL‐21), F4/80 (BM8), Siglec‐H (440c), CD19 (1D3), B220 (RAE‐6B2), Ly6G (1A8), MHCII (M5/114.15.2), and biotinylated anti‐SLAMF9 (M349). Secondary detection of SLAMF9 was performed using streptavidin conjugated to phycoerythrin or allophycocyanin. Instrumentation was performed on LSR Fortessa and LSR‐II flow cytometers (BD Biosciences). For all flow cytometry experiments, dead cells were excluded by either propidium iodide or fixable viability dyes from Thermo Fisher or BioLegend. Preliminary gating also excluded cellular aggregates by FSC‐A × FSC‐W or FSC‐A × FSC‐H analysis.

#### Primary macrophage and dendritic cell cultures

Human peripheral blood leucocyte cones from anonymous donors were obtained from the NHS Blood and Transplant Service. PBMCs were isolated by density gradient centrifugation using Histopaque 1077 (Sigma‐Aldrich), and monocytes were purified by magnetic enrichment using anti‐human CD14 magnetic beads according to the manufacturer's instructions (Miltenyi Biotech). Monocytes were plated in tissue culture flasks or multi‐well plates at a density of 1 × 10^5^/cm^2^ in RPMI‐1640 supplemented with 10% fetal calf serum (Sigma‐Aldrich), glutamax (Gibco, Grand Island, NY), Gibco MEM non‐essential amino acids (ThermoFisher) and penicillin/streptomycin (Sigma‐Aldrich), and the presence 50 U/ml granulocyte–macrophage colony‐stimulating factor (GM‐CSF), 100 U/ml macrophage colony‐stimulating factor (M‐CSF), or a combination of GM‐CSF and interleukin‐2 (IL‐4) 50 U/ml (all from Miltenyi Biotech). Four days after plating, media were supplemented with fresh cytokine at the levels indicated above. Cells were harvested for analysis after 7 days of differentiation. For generating mouse bone‐marrow‐derived macrophages, bone marrow from mice at least 8 weeks of age was harvested from tibia and femur by lavage and plated in 1 × 150‐cm^2^ tissue culture dishes per harvested leg. Cells were grown in Dulbecco's modified Eagle's medium supplemented with 10% fetal calf serum, glutamax, non‐essential amino acids and penicillin/streptomycin. For different macrophage types, media were supplemented with either 100 U/ml M‐CSF (Peprotech, Rocky Hill, NJ) or 50 U/ml GM‐CSF (Miltenyi Biotech). Cytokines were replenished in the media on day 4 after plating, and cells were harvested on day 7.

#### Stable knockdown of SLAMF9 using shRNA

A panel of SLAMF9‐specific short hairpin RNAs (shRNAs) in the lentiviral expression vector pLKO.1 were obtained from Open Biosystems (Dharmacon, Inc., Lafayette, CO). A non‐targeting shRNA control in pLKO.1 was a gift from John Sowerby. To generate viral particles, pLKO.1 targeting constructs, along with packaging vectors pMD2.G and pSPAX2 (gift from Didier Trono – Addgene plasmid numbers 12259 and 12260) were transfected into 293T cells. Lentivirus‐containing supernatants were collected after 72 hr and added to THP‐1 cells. Seventy‐two hours after transduction, cells were placed under puromycin (3 µg/ml) selection and maintained under selection during continuous culture. For differentiation of THP‐1 monocytes into macrophages, puromycin was removed from selection media, and cells were cultured in complete media containing 20 ng/ml phorbol 12‐myristate 13‐acetate (PMA). Expression levels of SLAMF9 were assayed by quantitative polymerase chain reaction (PCR) and flow cytometry. Clones TRCN0000142434 and TRCN0000143530 were found to provide strong suppression of SLAMF9 expression while maintaining cell viability. Further details on shRNA clones evaluated and antisense sequences are found in the Supplementary material (Table S1).

#### Measurement of cytokine production from THP‐1 cells

THP‐1 cells were obtained from ATCC (TIB‐202) and maintained in RPMI‐1640 supplemented with 10% fetal calf serum, sodium pyruvate, minimum essential medium non‐essential amino acids, glutamax, penicillin/streptomycin, and 50 µm 2‐mercaptoethanol. Before stimulation, THP‐1 cells were differentiated using 20 ng/ml PMA for 48 hr. Cells were then stimulated with 500 ng/ml LPS to stimulate pro‐inflammatory cytokine production. After 24 hr, cell‐free supernatants were collected and the indicated cytokines were measured by flow cytometry using Cytometric Bead Array Flex Sets (BD Biosciences). Measurements of statistical significance (unpaired *t*‐tests) were performed in graphpad prism v.6 software (GraphPad, San Diego, CA).

#### Quantitative RT‐PCR measurement of human SLAMF9 transcript expression

Total RNA from human THP‐1 cells, peripheral blood monocytes, and monocyte‐derived dendritic cells (DCs) was isolated using the RNeasy Mini Kit (Qiagen, Hilden, Germany). cDNA was prepared using SuperScript VILO cDNA synthesis kit. Quantitative PCR was performed on an ABI Prism 7900HT instrument using Thermo Fisher Scientific TaqMan probes: Hs01108640_g1‐FAM (SLAMF9); Hs00277217_m1‐FAM (SLAMF9); or Hs00824723_m1‐VIC (UBC).

#### SEC‐MALS determination of SLAMF9 solution properties

Soluble mouse SLAMF9 was produced in human HEK293T cells by lentiviral expression using the vector pLenti‐CMV‐GFP‐Puro (a gift from Eric Campeau; Addgene). Soluble SLAMF9 comprised the two immunoglobulin superfamily domains of SLAMF9 fused to rat CD4 domains 3+4 and a 6xHis tag in an AVEXIS‐ready configuration.^26^ Protein was expressed in FreeStyle 293 Expression medium and purified by Co^2+^ Immobilized Metal Ion Chromatography. Size exclusion chromatography coupled to multi‐angle light scattering (SEC‐MALS) was performed using a Wyatt Heleos II light scattering instrument and Wyatt Optilab rEX refractive index detector. The UV signal at 280 nm was collected with the Agilent 1200 SEC system. Data were analysed as described elsewhere using a dual detection (conjugate) method with refractive index increments for protein 0·186 1 g/ml and glycosylation 0·146 1 g/ml and with UV extinction coefficients for protein 1·28 ml/mg/cm and assuming no absorbance at 280 nm from glycosylation.^27^, ^28^

#### Immunoprecipitation, deglycosylation and Western blotting of SLAMF9

Native mouse SLAMF9 was immunoprecipitated from wild‐type or Slamf9^−/−^ bone‐marrow‐derived macrophages using biotinylated anti‐SLAMF9 (9318) and Dynal MyOne Streptavidin T1 magnetic beads (Thermo Fisher). Cells were lysed in phosphate‐buffered saline, 1% Triton‐X‐100 with Roche EDTA‐Free Protease Inhibitor Cocktail and lysates cleared by centrifugation before immunoprecipitation. Immunoprecipitated proteins were deglycosylated by treatment with NEB Protein Deglycosylation Mix according to the manufacturer's instructions before sodium dodecyl sulphate–polyacrylamide gel electrophoresis and Western transfer. Western blot detection of immunoprecipitated SLAMF9 was achieved using purified anti‐mouse SLAMF9 (9318) and anti‐rabbit IgG‐horseradish peroxidase (Cell Signaling Technologies, Danvers, MA).

#### *Salmonella* infection

Mice were screened for susceptibility to *Salmonella* infection as part of the efforts of the Infection, Immunity, and Immunophenotyping (3i) Consortium. Age‐ and sex‐matched C57BL/6N and *Slamf9^−/−^* mice 8–12 weeks in age were infected with 5 × 10^5^ colony‐forming units of *Salmonella enterica* Typhimurium strain M525 by intravenous injection. Mice were killed at days 14 and 28 post‐infection and liver and spleen were harvested for quantification of infectious burden. Bacteria from disaggregated spleen and liver were enumerated by serial dilution onto agar plates. Unpaired *t*‐tests for statistical significance were performed in graphpad prism v.6 software.

## Results

### Human monocytes up‐regulate SLAMF9 upon differentiation to macrophages or dendritic cells

**Figure 1:**
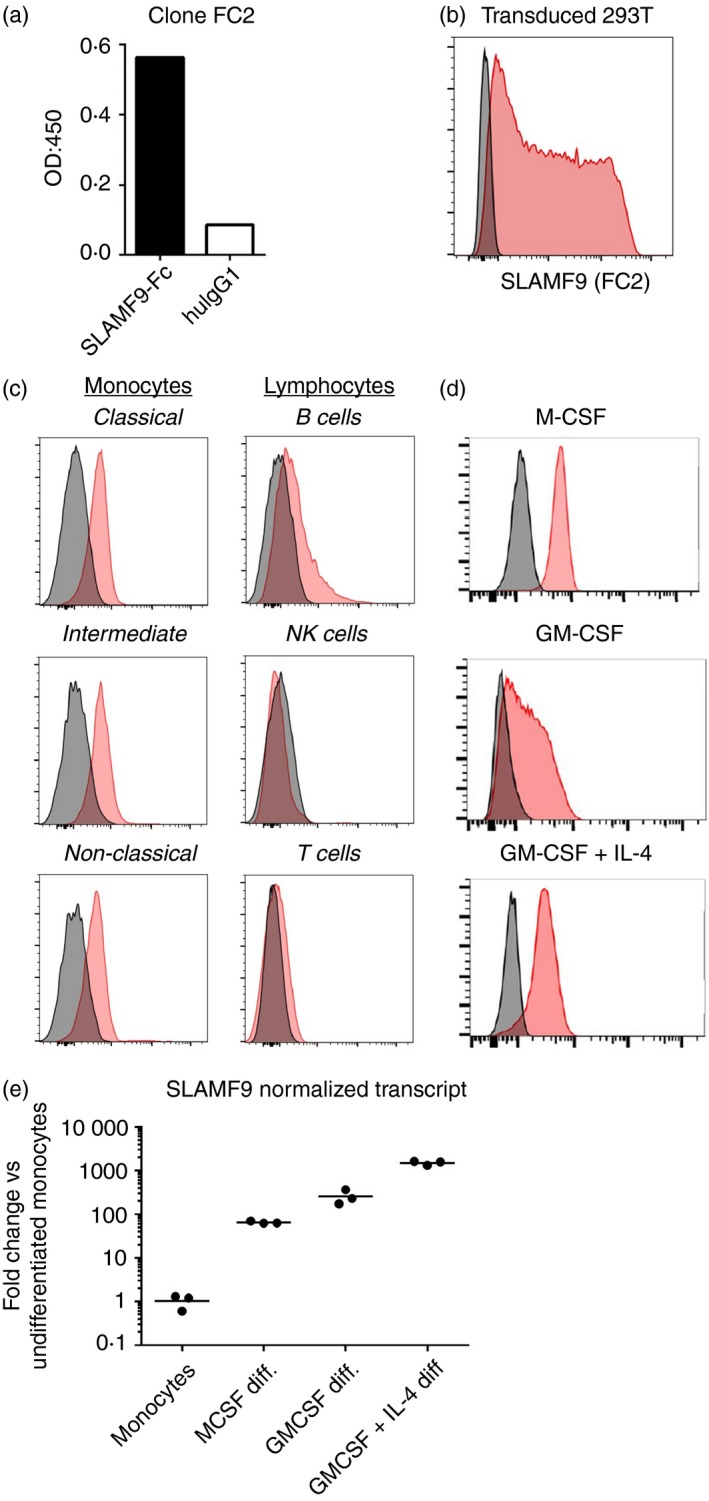
Human SLAMF9 is expressed on circulating monocytes and monocyte‐derived cells. (a) ELISA detection of antibodies produced by clone FC2 reactive to human SLAMF9‐Fc fusion protein but not human IgG1. (b) Flow cytometry validation of SLAMF9‐reactive hybridomas using HEK‐293T cells transduced with lentivirus encoding FLAG‐tagged human SLAMF9 (red) or untransduced cells (dark grey). (c, d) Surface expression of SLAMF9 (red) compared with isotype control (dark grey) measured by flow cytometry on freshly isolated PBMCs or monocyte‐derived cells. (c) Peripheral blood mononuclear cells (PBMCs) gated on: classical monocytes (CD14^+^ CD16^−^), intermediate monocytes (CD14^+^ CD16^+^), non‐classical monocytes (CD14^low^ CD16^+^), B cells (CD19^+^ FSC/SSC lymphocytes) natural killer (NK) cells (CD14^−^ CD16^+^, FSC/SSC lymphocytes), and T cells (CD3^+^, FSC/SSC lymphocytes). (d) Surface expression of SLAMF9 on CD14^+^ monocyte‐derived macrophages and dendritic cells differentiated for 7 days with the indicated cytokine(s). (e) Change in *SLAMF9* transcript expression during monocyte differentiation measured by quantitative RT‐PCR and normalized to *UBC.*

Since the original identification of human SLAMF9 in 2001, the cellular expression pattern of this receptor has remained largely undefined. Some anti‐human SLAMF9 antibodies have been published that work in Western blotting assays,^29^ but they have not been validated for recognition of native SLAMF9 on primary cells by flow cytometry. To determine the cellular expression of SLAMF9, monoclonal antibody reagents were developed to the extracellular domain of human SLAMF9. After initial ELISA‐based screening for hybridoma clones producing antigen‐reactive antibodies, clones were subsequently screened for reactivity to cell surface‐expressed SLAMF9 on transduced HEK293T cells. Clone FC2 was found to be reactive to soluble human SLAMF9‐IgG1‐Fc fusion protein (R&D Systems), but not human IgG1 (Sigma) by ELISA (Fig. 1a). Clone FC2 was further found to be specifically reactive to SLAMF9‐transduced HEK293T cells, but not untransduced controls (Fig. 1b). Using this new clone, cellular expression of SLAMF9 in human PBMCs from healthy donors was measured by flow cytometry and found to be largely restricted to monocytes, with low levels of expression found on all CD14‐ and CD16‐expressing monocyte subsets. Limited reactivity was also observed to some circulating B cells, with no reactivity observed to T cells or natural killer cells (Fig. 1c). Differentiation of monocytes into macrophages or DCs using M‐CSF, GM‐CSF, or a combination of GM‐CSF and IL‐4 led to more robust expression of SLAMF9 on the cell surface (Fig. 1d). This increased surface expression was coupled to a dramatic increase in *SLAMF9* transcript expression as measured by quantitative RT‐PCR (Fig. 1e).

### Production of pro‐inflammatory cytokines by THP‐1 cells is dependent on SLAMF9

**Figure 2:**
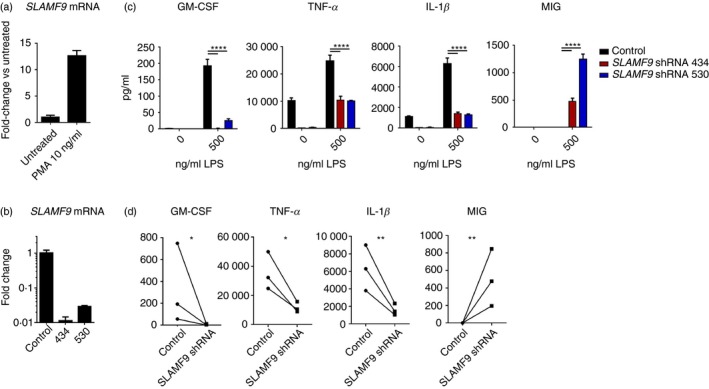
SLAMF9‐dependent activation of inflammatory cytokine production. (a) Quantitative RT‐PCR measurement of SLAMF9 expression in THP‐1 cells before and after differentiation with PMA. (b) Quantitative PCR measurement of SLAMF9 mRNA interference using stable expression of SLAMF9‐specific shRNAs (434 and 530) compared with a non‐targeting control shRNA in PMA‐differentiated THP‐1 cells. Transcript levels in (b) and (c) are normalized to *UBC*. (c) Pro‐inflammatory cytokine production by PMA‐differentiated THP‐1 cells with and without stimulation for 24 hr with lipopolysaccharide, measured by BD Cytometric Bead Array. Error bars indicate standard deviation from the mean. Statistical tests for differences between vector control and SLAMF9 knockdowns were performed using two‐way analysis of variance. ****P < 0·0001. (d) Analysis of cytokine expression in control and SLAMF9 knockdown (shRNA 434) THP‐1 cells across multiple independent experiments from (c). Ratio paired *t*‐tests show differences in cytokine production at *P < 0·05 and **P < 0·01.

Expression of *SLAMF9* transcript was previously reported in the human monocyte cell line, THP‐1.^18^ Expression of *SLAMF9* transcript in THP‐1 was examined by quantitative RT‐PCR at resting levels and following differentiation to macrophages using PMA or cytokines. Unlike primary monocytes, THP‐1 cells did not up‐regulate SLAMF9 in response to M‐CSF or GM‐CSF; however, SLAMF9 was found to be up‐regulated at the transcript level following PMA treatment of THP‐1 cells for 48 hr (Fig. 2a). To determine whether SLAMF9 could modulate the function of THP‐1 cells, SLAMF9 expression was targeted by RNA interference using stable expression of a SLAMF9‐specific shRNA in the lentiviral expression vector pLKO.1 (Open Biosystems, Dharmacon). Screening of multiple shRNA‐containing constructs yielded multiple effective shRNAs. Two were selected for further analysis based on knockdown efficiency and a lack of toxicity. These were clones TRCN0000142434 and TRCN0000143530, indicated by the numbers 434 and 530, respectively (Fig. 2b). THP‐1 lines stably expressing SLAMF9 shRNA or non‐targeting control shRNA were differentiated for 48 hr in PMA, then stimulated for 24 hr with *Escherichia coli* LPS to determine whether SLAMF9 affects pro‐inflammatory cytokine production. Indeed, SLAMF9 enhances the production of pro‐inflammatory cytokines GM‐CSF, tumour necrosis factor‐*α* (TNF‐*α*), and IL‐1*β* by THP‐1 cells. Interestingly, production of the chemokine MIG (CXCL9) was found to be suppressed by SLAMF9. This effect was observed first using shRNA 434 and confirmed with shRNA 530 (Fig. 2c). These data suggest that SLAMF9 could be an important contributor to pro‐inflammatory cytokine production in humans.

Since SLAMF9‐dependent changes to pro‐inflammatory cytokine expression were observed in THP‐1 cells following LPS stimulation without artificial cross‐linking of SLAMF9 on the cell surface, we hypothesize that PMA‐differentiated THP‐1 cells must simultaneously express SLAMF9 and a receptor/ligand for this protein. As all other CD2/SLAM family receptors are either homophilic or interact with other members of the CD2 family, we screened for interactions between SLAMF9 and members of the CD2 family. Production of soluble human SLAMF9 was unsuccessful, most likely because of intrinsic instability of the protein. In contrast, all mouse CD2‐family members, including SLAMF9, were readily produced as soluble CD4‐fusion proteins according to previously described methods.^26^ SLAMF9 was screened against all mouse CD2/SLAM family receptor proteins by surface plasmon resonance (SPR) using a Biacore T200 system. We failed to observe reproducible interactions between mouse SLAMF9 and any member of the CD2 family (not shown). Since homotypic interactions can be difficult to observe and quantify by SPR, the soluble mouse SLAMF9 was examined using SEC‐MALS to detect complexes in solution. The use of a dual detection (conjugate) method employing refractive index and UV as measures of concentration indicated that the mouse SLAMF9‐CD4 construct was predominantly monodisperse material of total mass 58 000 comprising 43 000 protein with 15 000 post‐translational glycan modification (see Supplementary material, Fig. S1A). This is in close agreement with theoretical values based on sequence data of 44 300 protein mass and six N‐linked glycosylation sites with an expected average MW of 2500 each for a total of 15 000 expected sugar mass. These values did not change over a wide range of concentrations (~1–20 μm) of SEC‐MALS measurement indicating that SLAMF9 is predominantly monomeric under these conditions (see Supplementary material, Fig. S1). These biochemical analyses indicate that mouse SLAMF9 is unlikely to be homophilic and remains an orphan receptor within the CD2/SLAM family of receptors.

### Mouse SLAMF9 has restricted expression among antigen‐presenting cells

**Figure 3:**
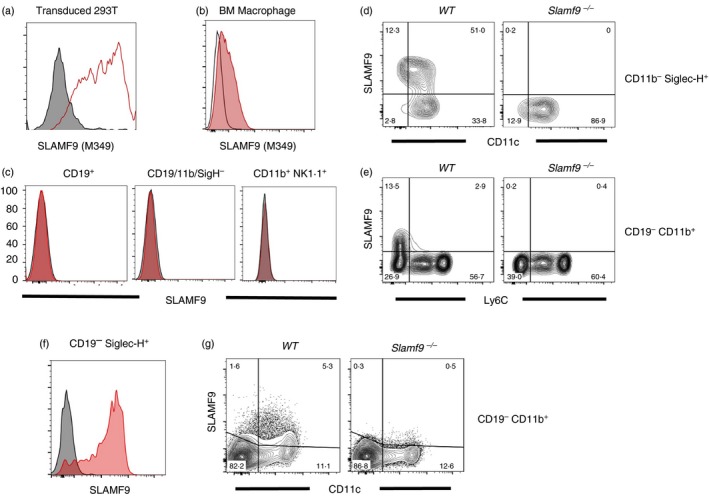
Mouse SLAMF9 is expressed on circulating plasmacytoid dendritic cells (pDCs) and non‐classical monocytes. (a) Flow cytometry screening of mouse SLAMF9 reactive hybridomas against HEK‐293T cells lentivirally transduced to express mouse SLAMF9 (red line) or untransduced (shaded) cells. (b) Validation of mSLAMF9‐reactive hybridoma clone M349 on mouse bone marrow‐derived macrophages from wild‐type C57BL/6 (red shaded) or *Slamf9^−/−^* mice (black line). (c–e) Flow cytometry staining of SLAMF9 on circulating peripheral blood leucocytes using anti‐SLAMF9 clone M349 with cells from *Slamf9^−/−^* mice used as a negative staining control. (c) Staining of WT C57BL/6 (red) or *Slamf9^−/−^* (grey) B cells, T cells and natural killer cells shows an absence of SLAMF9 on resting lymphocytes. (d) SLAMF9 staining is found on CD11b^–^ Siglec‐H^+^ CD11c^low^ pDCs and (e) CD19^−^ CD11b^+^ Ly6C^−^ non‐classical monocytes. (f,g) Flow cytometry staining of mouse splenocytes using anti‐SLAMF9 antibody M349 finds expression of SLAMF9 on (f) wild‐type (red) but not knockout (grey) CD19^−^ Siglec‐H^+^ pDCs and (g) a fraction of CD11b^+^ CD11c^+^ cDCs.

To understand the mechanism by which SLAMF9 contributes to immunity in mice, it was first necessary to determine the cellular expression pattern of SLAMF9 on leucocytes. As the cellular expression of SLAMF9 is incompletely characterized and validated monoclonal antibodies were not commercially available when this study commenced, monoclonal antibodies to mouse SLAMF9 were generated by immunizing *Slamf9^−/−^* mice with soluble mouse SLAMF9. Clones identified as SLAMF9‐reactive by ELISA were subsequently screened by flow cytometry on mSLAMF9‐expressing HEK293T cells and on mouse bone‐marrow‐derived macrophages. Hybridoma clone M349 was selected for further characterization (Fig. 3a,b).

Examination of mouse PBMCs found no SLAMF9 expression on circulating CD19^+^ B cells, NK1.1^+^ natural killer cells, or CD11b^–^, CD19^–^, NK1.1^–^ lymphocytes, including T cells and innate lymphoid cell falling in this gate. (Fig. 3c). SLAMF9 expression in PBMCs is found in CD19^−^ CD11b^−^ CD11c^low^ Siglec‐H^+^ plasmacytoid DCs (pDCs) (Fig. 3d) and in a fraction of CD11b^+^ Ly6C^–^ non‐classical monocytes (Fig. 3e). Expression of SLAMF9 in spleen is similar to peripheral blood, with abundant expression of SLAMF9 in pDCs (Fig. 3f). The expression of SLAMF9 by pDCs was corroborated by recent literature.^30^ Among CD11b^+^ cells, weak expression of SLAMF9 is found in CD11c^high^ conventional DCs, whereas highest expression of SLAMF9 in myeloid cells was found among a fraction of CD11c^low^ cells corresponding to the Ly6C^–^ monocyte subset observed in blood (Fig. 3g).

**Figure 4:**
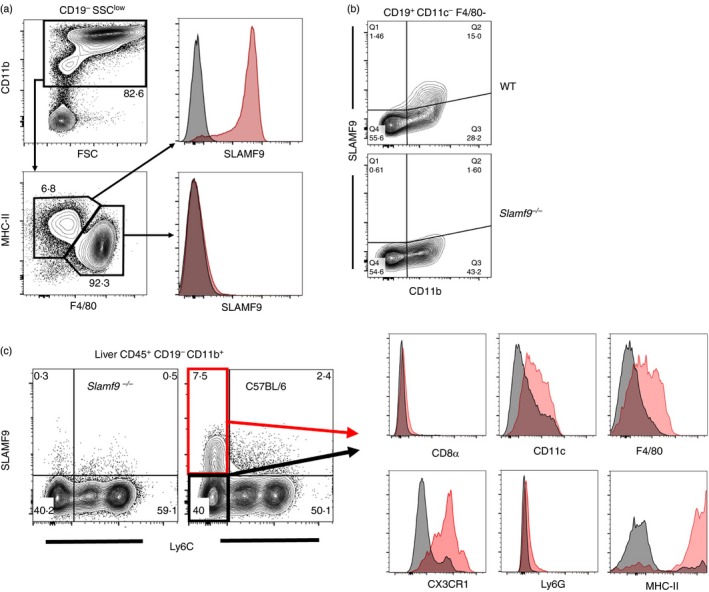
SLAMF9 in mouse tissues is found on niche antigen‐presenting cells. Staining of live, single cells from peritoneal lavage for SLAMF9 expression. Cells from *Slamf9−/−* mice are used as a negative staining control for SLAMF9 expression found on wild‐type C57BL/6 cells. SLAMF9 is restricted to (a) CD19^−^ CD11b^int^ MHC‐II^+^ F4/80^low^ small peritoneal macrophages and (b) CD19^+^ CD11b^+^ B1 cells, but is absent on the more abundant CD11b^high^ F4/80^high^ large peritoneal macrophages and CD19^+^ CD11b^−^ B2 cells. (c) Expression of SLAMF9 on leucocytes from perfused and collagenase‐disaggregated liver is also restricted to PDCs (not shown) and a subset of CD11b^+^ Ly6C^−^ mononuclear phagocytes. Gating on SLAMF9^+^ (red histogram) and SLAMF9^−^ (black histogram) CD11b^+^ Ly6C^−^ cells shows a surface phenotype for SLAMF9^+^ cells as CD11c^+^ F4/80^low^ CX_3_CR1^+^ MHC‐II^high.^

In keeping with a restricted cellular expression pattern in blood and tissues, SLAMF9 in the peritoneal cavity is abundantly expressed by CD11b^+^ MHC‐II^+^ F4/80^low^ small peritoneal macrophages^31^ but is absent on resting CD11b^high^ F4/80^high^ large peritoneal macrophages (Fig. 4a). SLAMF9 is also present on a fraction of CD19^+^ CD11b^+^ B1 cells (Fig. 4b), but absent on all peritoneal CD19^+^ CD11b^−^ B2 cells and CD11b^−^ cells. This expression pattern implicates SLAMF9 in induced inflammatory responses following infectious stimulus.^32^

As SLAMF9 in humans is up‐regulated upon differentiation of blood monocytes into macrophages or DCs (Fig. 1d,e), we wanted to examine whether SLAMF9 in mice would be more abundant on tissue‐resident populations of mononuclear phagocytes. To examine this possibility, mouse livers were perfused through the portal vein with HBSS containing collagenase D to remove blood and aid tissue disaggregation. Leucocytes were then enriched by Percoll‐gradient centrifugation and examined by flow cytometry. As with blood, SLAMF9 expression in liver was restricted to pDCs (not shown) and CD11b^+^ Ly6C^–^ mononuclear phagocytes. Further examination of the surface marker phenotype of liver CD11b^+^ Ly6C^–^ SLAMF9^+^ cells found them to be CD11c^low^, CD8α^–^, F4/80^low^, CX_3_CR1^+^, Ly6G^−^ and MHC‐II^high^ (Fig. 4c). This implicates patrolling monocytes or tissue‐resident type 2 conventional DCs as the likely source of CD11b^+^ SLAMF9^+^ cells in resting liver.^33^, ^34^

### SLAMF9 promotes resistance to systemic *Salmonella* infection

**Figure 5:**
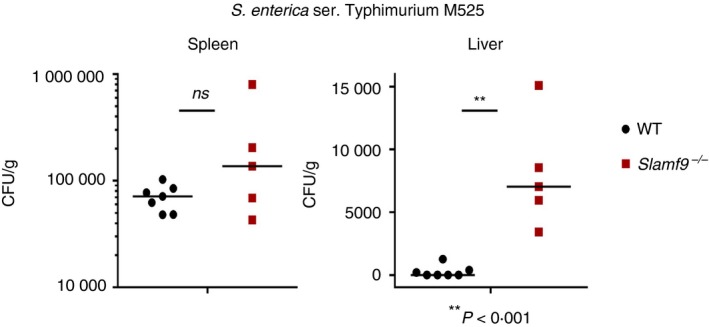
SLAMF9 promotes resistance to *Salmonella*. C57BL/6 and *Slamf9^−/−^* mice were infected with *Salmonella enterica* serovar Typhimurium M525 by intravenous injection. Spleen and liver were harvested at day 14 post‐infection and CFU/g was quantified. Each dot represents a single mouse. *P*‐values were determined using Student's *t*‐test. Results are representative of two independent experiments.

*Slamf9^−/−^* mice were generated by homologous recombination and examined for major defects in the number and type of leucocytes within central and peripheral lymphoid organs. No major defects in lymphoid or myeloid cell development were observed within bone marrow, thymus, spleen, lymph nodes, peritoneal cavity or liver (not shown). To examine immune responses to infection, wild‐type and *Slam9^−/−^* mice were given systemic infection with *S. enterica* serovar Typhimurium M525 by intravenous injection. After 14 days, mice were killed and the total bacterial burden in spleen and liver were quantified. Although bacterial burden in *Slamf9^−/−^* spleens was comparable to that in wild‐type C57BL/6N controls, *Slamf9^−/−^* mice were impaired in their ability to clear *Salmonella* from the liver (Fig. 5). This demonstrates a role for SLAMF9 in the resistance to systemic infection with *Salmonella*.

## Discussion

The initiation of inflammation is very important to generating protective immunity following infection. Although a great deal is understood about the recognition of microbial products by pattern recognition receptors, less is understood about how other cell surface receptors may influence the subsequent signalling cascades. In this study, we have shown that SLAMF9‐deficient mice have impaired clearance of systemic *Salmonella* infection, and suppression of SLAMF9 expression in human THP‐1 cells dramatically reduces their ability to produce pro‐inflammatory cytokines TNF‐*α*, IL‐1*β* and GM‐CSF. This indicates that SLAMF9 is essential for optimum production of these cytokines in response to LPS detection and may indicate a broader role for SLAMF9 in the initiation of inflammation. These data are corroborated by recent results showing that overexpression of SLAMF9 in U937 cells can augment their TNF‐*α* production in response to LPS.^22^ It is interesting to note that the knockdown of SLAMF9 did not result in a global decrease in cytokine production, because other cytokines such as IL‐6 were not significantly different (not shown), and production of the chemokine CXCL9 (MIG) was induced in response to LPS only when SLAMF9 expression was suppressed. This suggests that SLAMF9 may not simply potentiate TLR signalling, but instead influences which genes are expressed downstream of TLR activation. The question of whether SLAMF9 is primarily influencing phagocyte activation or differentiation will be important for understanding the mechanism by which SLAMF9 contributes to *Salmonella* resistance.

A recent report has implicated SLAMF9 in the development of mouse pDCs, with a mild impairment in pDC maturation in the absence of SLAMF9 leading to the accumulation of immature pDCs in lymph nodes and a reduction in the percentage of interferon‐*α*‐producing pDCs after CpG ODN stimulation.^30^ Type I interferon signalling is known to contribute to macrophage necroptosis during *Salmonella* infection.^35^ Therefore, we might have expected enhanced clearance of *Salmonella* in *Slamf9^−/−^* mice. However, Sever et al. also reported a relative decrease in the frequency of TNF‐*α*‐producing pDCs in CpG‐stimulated lymph node cells from *Slamf9^−/−^* mice with experimental autoimmune encephalomyelitis.^30^ If optimal TNF‐*α* production is dependent on SLAMF9 expression across multiple cell types, this may help to explain the impaired clearance of *Salmonella* in our studies.

Although SLAMF9 is important for generating inflammatory responses, the mechanism by which SLAMF9 senses the extracellular environment and delivers a signal is unknown. All SLAM family receptors except for SLAMF9 have known ligands. CD48 and 2B4 interact heterotypically with one another,^36^, ^37^ whereas SLAM,^38^ Ly9,^9^ CD84,^39^ NTB‐A,^12^, ^40^ CRACC,^10^, ^13^ and BLAME^11^ are homophilic. References to putative homotypic interactions by SLAMF9 are found in the literature,^21^, ^41^ but the origins of this assumption are unclear, because no published data exist showing self‐association by SLAMF9. In our studies on mouse SLAMF9, no evidence for self‐association was observed by SEC‐MALS (see Supplementary material, Fig. S1) or SPR (not shown). We conclude that mouse SLAMF9 is unlikely to be homophilic. We were also unable to find reproducible binding of SLAMF9 to any other CD2/SLAM family receptors by SPR. These observations lead us to the conclusion that any ligand for SLAMF9 is most likely outside the CD2 family.

We found no data to support the notion that SLAMF9 is homophilic in mice, but we cannot rule out homotypic interactions of SLAMF9 in other species, such as humans. In comparison with mouse SLAMF9, human SLAMF9 is difficult to produce as a soluble protein and is expressed at low levels on the surface of monocyte‐derived phagocytes or transfected fibroblasts. This limited our ability to test for protein–protein interactions by human SLAMF9. Knockdown of SLAMF9 expression in PMA‐differentiated human THP‐1 cells produced a remarkable reduction in pro‐inflammatory cytokine production following LPS stimulation. As no artificial cross‐linking of SLAMF9 was performed in this assay, any extracellular ligand for SLAMF9 was already present in the experimental system. This could be interpreted as supporting evidence for self‐association; however, given the absence of homotypic interactions by mouse SLAMF9, it is very likely that SLAMF9 is interacting with another protein on the surface of THP‐1 cells or within the serum used for cell culture.

SLAMF9 is important for pro‐inflammatory cytokine production, but the means by which SLAMF9 delivers a pro‐inflammatory signal is unclear. Since SLAMF9 lacks any ITSMs or other recognizable cytoplasmic signalling motifs, it is possible that it simply serves as a ligand for another receptor that has signalling capability. Alternatively, it may deliver a signal through association with adapter molecules. Other SLAM family members without cytoplasmic ITSMs are able to signal without direct modification by tyrosine kinases. Although CD48 functions as the ligand for CD244 (2B4/SLAMF4), it can signal through its association with lipid rafts,^42^ and homotypic interactions by BLAME influence cellular function through unknown mechanisms.^11^, ^17^, ^21^

A recent report suggests that BLAME and SLAMF9 function redundantly to modulate inflammation in the liver through indirect regulation of TLR4 expression,^21^ but we found impaired clearance of *Salmonella* from the liver without additional modulation of BLAME. So although BLAME and SLAMF9 may have some overlapping function, SLAMF9 functions distinctly from BLAME to promote inflammation and immunity. Furthermore, we find a unique expression pattern for SLAMF9. While SLAMF9 expression has been observed on macrophages,^21^, ^22^ it is not found universally or exclusively on macrophage subsets. Looking at the single cell level using flow cytometry, we find SLAMF9 on B1 cells, pDCs, small peritoneal macrophages, and a fraction of tissue phagocytes that are CD11b^+^ Ly6C^−^ CD11c^low^ and CX_3_CR1^+^. These may include activated patrolling monocytes^43^ or a subset of cDC2 cells.^34^ These data using the M349 monoclonal antibody generated for this study are consistent with transcript data published by the Immunological Genome Project and are largely distinct from the reported expression patterns for BLAME, making functional redundancy of the two receptors unlikely at the cellular level. Like many other cell surface receptors, mouse SLAMF9 is heavily glycosylated (see Supplementary material, Fig. S1B), with six potential N‐linked glycosylation sites fitting the canonical NxT or NxS motif in its extracellular domains. For this reason, migration on sodium dodecyl sulphate–polyacrylamide gel electrophoresis gels is reduced and mature SLAMF9 from bone marrow‐derived macrophages appears as a heterogeneous species at a much higher molecular weight (40 000–70 000) than predicted by peptide sequence alone (30 000) on Western blots. Therefore, caution should be used when interpreting results where a single band at approximately 30 000 on a Western blot has been used to validate other mouse SLAMF9‐reactive antibodies.

In summary, we have identified SLAMF9 as an important mediator of inflammation and a contributor to antibacterial immune responses to systemic *Salmonella* infection. Further studies on the mechanism by which SLAMF9 mediates these functions will be important for the biology of mononuclear phagocytes as well as evaluation of SLAMF9 as a potential therapeutic target for modulating inflammatory responses.

## Author contributions

TJW conducted experiments, generated critical reagents, directed research and wrote/edited the manuscript. SC provided critical reagents and directed research. JM and KH conducted experiments; CMJ conducted experiments and edited the manuscript; PAL directed research; GD provided critical reagents and directed research; and KGCS directed research and edited the manuscript.

## Disclosure

The authors declare no competing financial interests.

## Supporting information


**Figure S1.** (A) SEC‐MALS analysis of soluble mouse SLAMF9‐CD4 fusion protein shows a modified, monodisperse protein sample of approximate protein mass, 43 000 (expected monomer: 46 000), indicating mouse SLAMF9 is not homophilic. (B) Immunoprecipitation and Western blotting of mouse SLAMF9 from wild‐type and *Slamf9^−/−^* bone‐marrow‐derived macrophages using rabbit anti‐mouse SLAMF9 (Pab 9318). Immunoprecipitation eluates are blotted with and without sample treatment with exoglycosidases.

**Figure S2.** Tumour necrosis factor‐*α* (TNF‐*α*) production from mouse bone‐marrow‐derived macrophages. Mouse macrophages derived from wild‐type and *Slamf9^−/−^* bone marrow cultured for 7 days in M‐CSF were stimulated with 500 ng/ml lipopolysaccharide and cytokines were assayed by cytometric bead array. TNF‐*α* production (measured in technical duplicate or triplicate) from three independent experiments is shown. Significant changes in TNF‐*α* production were not reproducibly observed.

**Table S1.** Lentiviral short hairpin RNA clones used for RNA interference in THP‐1.
