# Supplementary figures and images for: Signalling lymphocyte activation molecule family member 9 is found on select subsets of antigen‐presenting cells and promotes resistance to Salmonella infection
Source: Immunology. 2020 Jan 28;159(4):393–403. doi: 10.1111/imm.13169 (PMC7078004; doi:10.1111/imm.13169)

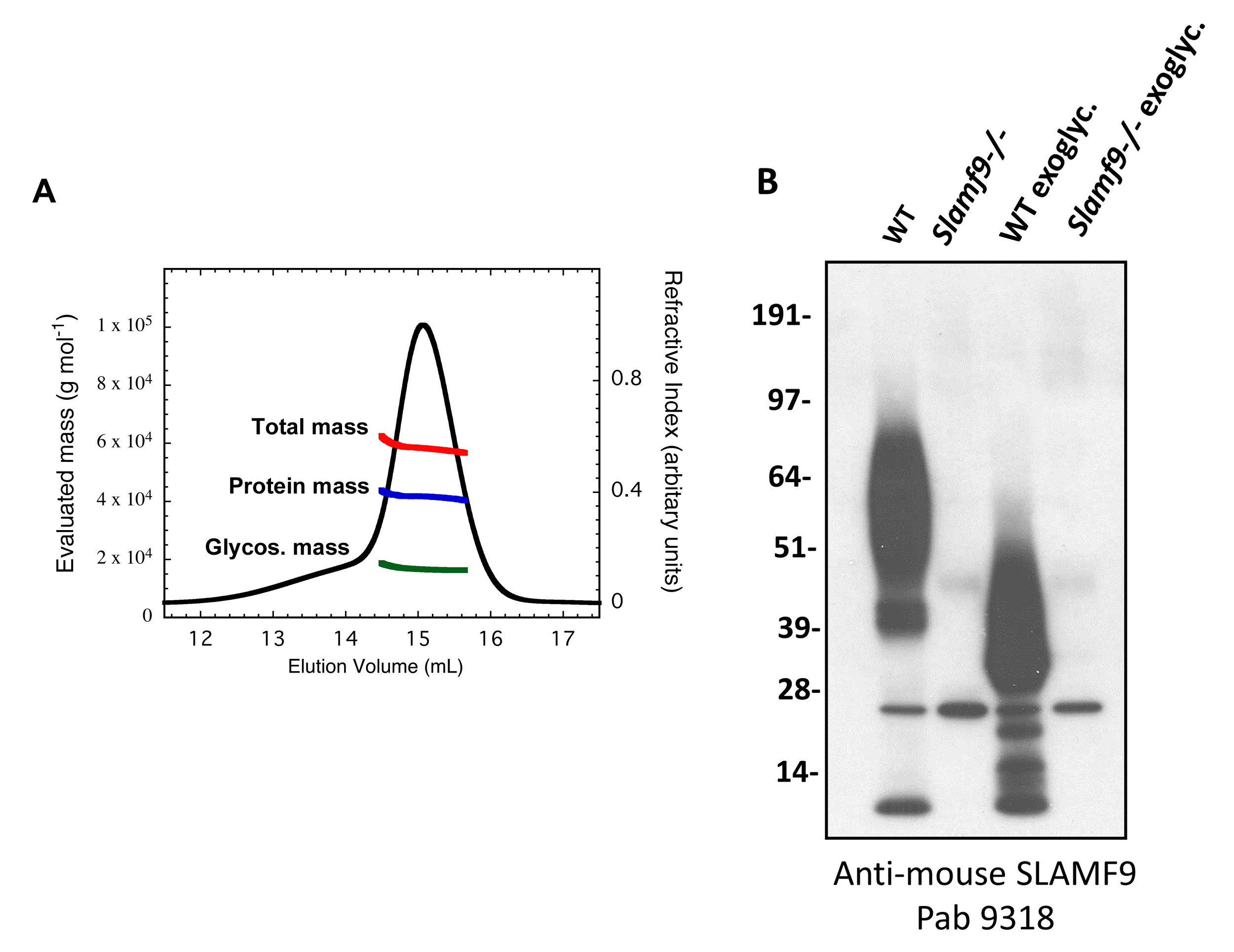

Supplement: Supplementary file 1 — Figure S1. (A) SEC‐MALS analysis of soluble mouse SLAMF9‐CD4 fusion protein shows a modified, monodisperse protein sample of approximate protein mass, 43 000 (expected monomer: 46 000), indicating mouse SLAMF9 is not homophilic. (B) Immunoprecipitation and Western blotting of mouse SLAMF9 from wild‐type and Slamf9−/− bone‐marrow‐derived macrophages using rabbit anti‐mouse SLAMF9 (Pab 9318). Immunoprecipitation eluates are blotted with and without sample treatment with exoglycosidases. [file IMM-159-393-s001.jpg]

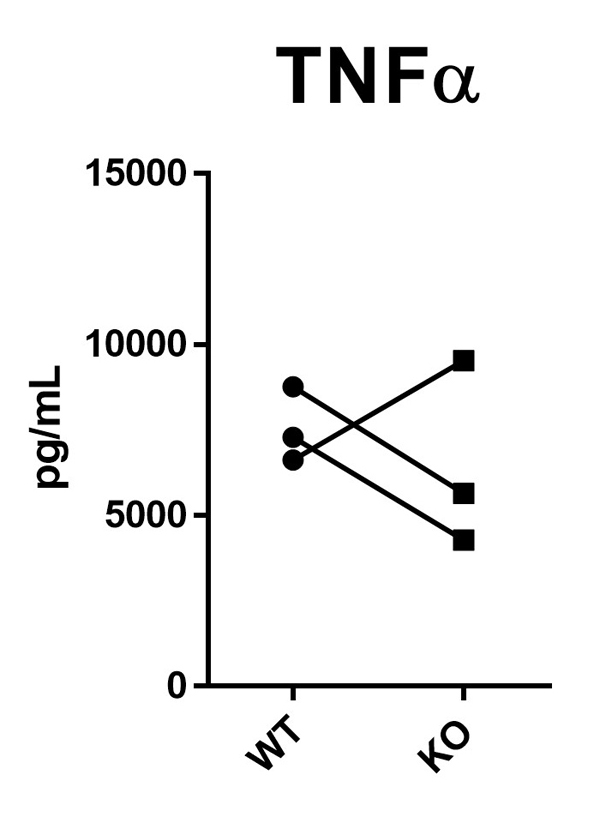

Supplement: Supplementary file 2 — Figure S2. Tumour necrosis factor‐α (TNF‐α) production from mouse bone‐marrow‐derived macrophages. Mouse macrophages derived from wild‐type and Slamf9−/− bone marrow cultured for 7 days in M‐CSF were stimulated with 500 ng/ml lipopolysaccharide and cytokines were assayed by cytometric bead array. TNF‐α production (measured in technical duplicate or triplicate) from three independent experiments is shown. Significant changes in TNF‐α production were not reproducibly observed. [file IMM-159-393-s002.jpg]
