# Supplementary material for: Signalling lymphocyte activation molecule family member 9 is found on select subsets of antigen‐presenting cells and promotes resistance to Salmonella infection
Source: Immunology. 2020 Jan 28;159(4):393–403. doi: 10.1111/imm.13169 (PMC7078004; doi:10.1111/imm.13169)
Supplement: Supplementary file 3 — Table S1. Lentiviral short hairpin RNA clones used for RNA interference in THP‐1. [file IMM-159-393-s003.docx]

| **Supplementary Table 1**: Lentiviral shRNA clones used for RNA interference in THP-1. | | |
| --- | --- | --- |
| **SLAMF9 shRNA** | **Mature antisense** | **Percent Transcript Expression** |
| TRCN0000142434 | AUGAGUUUCUUCAUCCUUGGC | 2.7% |
| TRCN0000141907 | AGAAGCAUAGUUAGGAUCUGC | 17.6% |
| TRCN0000140086 | AUGGCCAGAAUUACCAAGAGC | 14.7% |
| TRCN0000140890 | AGCUGUAGGUCAUAUCCAUGC | 7.7% |
| TRCN0000143530 | AUGACACAUAGAUUGUACUGC | 8.1% |
| Non-targeting control | ACCUAAGGGCUAAGAGCGCAC | 100% |
